# Essential Tremor and Mortality in Older Adults: The Role of Word Recall, a Measure of Episodic Memory, in a 23-Year Follow-Up Study

**DOI:** 10.3390/jcm14041160

**Published:** 2025-02-11

**Authors:** Julián Benito-León, José Lapeña-Motilva, Ritwik Ghosh, Verónica Giménez de Béjar, Carla Mª Benito-Rodríguez, Félix Bermejo-Pareja

**Affiliations:** 1 Department of Neurology, 12 de Octubre University Hospital, 28041 Madrid, Spain; joselapemo@gmail.com; 2 Instituto de Investigación Sanitaria Hospital 12 de Octubre (imas12), 28041 Madrid, Spain; neuro.gimenezdebejar@gmail.com (V.G.d.B.); felixbermejo006@gmail.com (F.B.-P.); 3 Centro de Investigación Biomédica en Red Sobre Enfermedades Neurodegenerativas (CIBERNED), 28031 Madrid, Spain; 4 Department of Medicine, Faculty of Medicine, Complutense University, 28040 Madrid, Spain; 5 Department of General Medicine, Burdwan Medical College and Hospital, Burdwan 713104, WB, India; ritwikmed2014@gmail.com; 6 Faculty of Medicine, University of Alfonso X el Sabio, Villanueva de la Cañada, 28691 Madrid, Spain; carlaabeenito@gmail.com

**Keywords:** essential tremor, older adults, episodic memory, NEDICES cohort, mortality risk: cognitive impairment, population-based survey

## Abstract

**Background:** The association between essential tremor (ET) and mortality risk remains uncertain. This study investigated the impact of episodic memory performance, measured through a word recall task, on mortality risk in ET within the Neurological Disorders in Central Spain (NEDICES) cohort, a population-based study of older adults. **Methods**: Participants were followed until death or 31 December 2017, and divided into four groups based on ET status and memory performance (errors in the 37-Minimental Examination’s three-word recall task). Cox proportional hazards models estimated mortality hazard ratios (HRs), and the Relative Excess Risk due to Interaction (RERI) assessed additive interactions. **Results**: Among 3998 participants, 3432 (85.8%) died over a median follow-up of 11.2 years. ET patients with episodic memory impairments had a higher mortality risk (HR = 1.25, 95% CI: 1.06–1.46) compared with controls with similar deficits (HR = 1.19, 95% CI: 1.09–1.28), whereas no significant increase was observed for ET patients without memory impairments (HR = 0.95, 95% CI: 0.74–1.21). RERI analysis revealed no significant additive interaction between ET and memory impairment (fully adjusted RERI: 0.11 [95% CI: −0.19–0.41]). Episodic memory impairments, regardless of ET status, were strongly associated with Alzheimer’s disease as a primary cause of death. **Conclusions**: These findings highlight the independent contribution of episodic memory impairment to increased mortality risk, with ET modestly amplifying this effect without significant interaction. Further research is needed to explore shared pathophysiological mechanisms between ET and neurodegenerative conditions.

## 1. Introduction

There is growing evidence that suggests a compelling link between cognitive impairment and dementia, including Alzheimer’s disease (AD), and increased mortality risks in older people [1,2,3,4]. A critical aspect of this link is the role of episodic memory impairment, which is a hallmark of AD, one of the earliest domains to decline and related to an increased risk of mortality [1,5].

In large population studies, extensive episodic memory testing is impractical due to the considerable time required. Therefore, alternative methods that offer a quicker and more practical approach to evaluating episodic memory are essential in these research contexts. Given these constraints, the simplicity of the Mini-Mental State Examination’s (MMSE) three-word recall task emerges as a more practical tool. This task offers a faster and more efficient way to measure episodic memory, particularly in large-scale studies [1,5,6]. Also, its proven ability to predict mortality and dementia offers a practical yet effective tool for large-scale epidemiological studies [1,5]. In the MMSE’s three-word recall task, the patient is asked to remember and later recall three unrelated words.

ET patients generally have poor performance on language, verbal and visual memory, and frontal executive function tests [7,8,9], and these cognitive changes are associated with more functional difficulty [10]. These changes are not static and can worsen significantly [11,12]. Specifically, several extensive epidemiological studies have demonstrated an association between ET with mild cognitive impairment and dementia [11,12,13,14,15,16,17,18], including AD [14,15]. A postmortem investigation revealed that ET patients accumulate amyloid-β in the cerebellar cortex [19]. In another study, cognitively unimpaired ET patients and those with mild cognitive impairment had more hyperphosphorylated forms of the microtubule-associated protein tau in neurofibrillary tangles in the neocortex than their non-ET counterparts [20]. These findings suggest a possible link between ET and the neuropathological hallmarks of AD, highlighting the need for further research into the neurological underpinnings and potential cognitive implications of ET. In addition, the risk of incident dementia was found to be higher in individuals with ET in two separate population-based studies (one in Spain and the other in New York) [15,16].

A significant gap remains in our understanding of the relationship between ET and mortality risk. Only a paucity of studies (especially prospective population-based studies) explicitly focused on the mortality risk among ET patients [21,22,23,24]. Given that cognitive impairments in ET, particularly in episodic memory, mirror those observed in the early stages of AD [7,8,9,25,26], further research is needed to explore potential links between ET, cognitive decline, and mortality risk.

We aimed to bridge this gap using the Neurological Disorders in Central Spain (NEDICES) cohort. By focusing on the MMSE-37’s three-word recall task to assess episodic memory in older adults with ET [1,5,6], we sought to elucidate the relationship between episodic memory impairment and mortality risk in this population. This study tested two hypotheses: firstly, that poorer episodic memory performance in ET patients correlates with a higher mortality risk, and secondly, that ET patients with episodic memory impairments are at an increased risk of mortality from AD.

## 2. Methods

### 2.1. Study Population

The data for the current studies were obtained from the NEDICES study, a comprehensive, community-based research project examining the prevalence, incidence, and factors influencing primary conditions related to aging in the older population [27,28,29]. Detailed accounts of the study population and sampling methods have been published elsewhere [27,28,29].

### 2.2. Study Evaluation

During the initial (1994–1995) and follow-up (1997–1998) assessments, participants were interviewed with a questionnaire designed to gather information on demographic factors, medication use, including drugs with potential cognitive effects such as anxiolytics, stimulants, antipsychotics, antidepressants, antihistamines, antihypertensives, and antiepileptics, medical history, smoking (ever vs. never), and drinker (ever/at least once per week vs. never) [27,28,29]. A short questionnaire was mailed to subjects who refused or were unavailable for face-to-face interviews.

A 37-item Mini-Mental State Examination (MMSE-37) was administered to assess global cognition. This test is a Spanish adaptation of the standard MMSE [30,31], which essentially follows the original procedure of Folstein et al. [32]. The delayed free recall task was conducted immediately following the attention assessment. The interval between registration and free recall tasks was about three minutes [30,31].

According to a comorbidity score developed in ambulatory care settings [33], a comorbidity index was calculated [33]. Each participant was also asked to indicate their total hours of actual sleep in 24 h (sum of nighttime sleep and daytime napping) [34,35].

One screening question for ET was included in the baseline (1994–1995) and follow-up assessment (1997–1998): “Have you ever suffered from a tremor of the head, hands, or legs that has lasted longer than several days?” [36,37].

Participants were considered a positive screening for ET if they answered “yes” to the screening question for ET [36,37]. The sensitivity of this screening question was evaluated by selecting and contacting a random sample of approximately 4% of subjects who had screened negative [38]. Each of these subjects subsequently underwent a neurologic examination [38]. During the neurologic examination, none of the 183 participants who tested negative at screening were found to have ET (sensitivity: 100%; negative predictive value: 100%) [38].

At baseline and follow-up [36,37], participants who screened positive for ET underwent a neurologic evaluation by senior neurologists. The participants’ medical records, which were unavailable for assessment, were sourced from various places. ET cases were initially identified by one neurologist and subsequently examined by two additional neurologists. Patients were classified as having ET only when all three neurologists reached a consensus, thereby minimizing the likelihood of initial diagnostic errors. The same rigorous methodology was applied during the second assessment (1997–1998) to ensure consistency in diagnostic criteria over time.

The diagnostic criteria for ET in this study, which were applied to both direct examinations and reviews of participants’ medical records, were derived from the criteria used in the Sicilian Study [39,40]. ET diagnosis was confirmed in participants exhibiting action tremors of the limbs or head, with no attributable alternative causes.

Among the 5278 participants screened for ET from 1994 to 1995, 256 prevalent cases (4.8%) were identified [36]. This prevalence rate aligns closely with other population-based estimates of ET prevalence in Western countries, underscoring the validity of the diagnostic criteria used in this study [41].

We used the Diagnostic and Statistical Manual of Mental Disorders, Fourth Edition criteria to diagnose dementia [42,43,44].

### 2.3. Mortality Data

Mortality data of the cohort were obtained until 31 December 2017. The Spanish National Population Register (*Instituto Nacional de Estadística* in Spanish) provides the death dates. In Spain, the death certificates are issued by a physician at the time of death and then sent to the local municipal authority where the deceased resided, allowing the information to be recorded in the National Population Register. Death certificates issued by attending physicians were categorized using ICD-10 codes, with cases initially coded under ICD-9 recoded to ICD-10 by neurologists and a statistician to ensure consistency. The principal underlying cause of death was determined as the condition initiating the sequence leading to death. In cases where the death certificates specified dementia according to ICD-10th criteria, we conducted an in-depth review if there was a pre-existing clinical diagnosis of AD confirmed by NEDICES neurologists.

### 2.4. Final Selection of Study Participants

**Figure 1:**
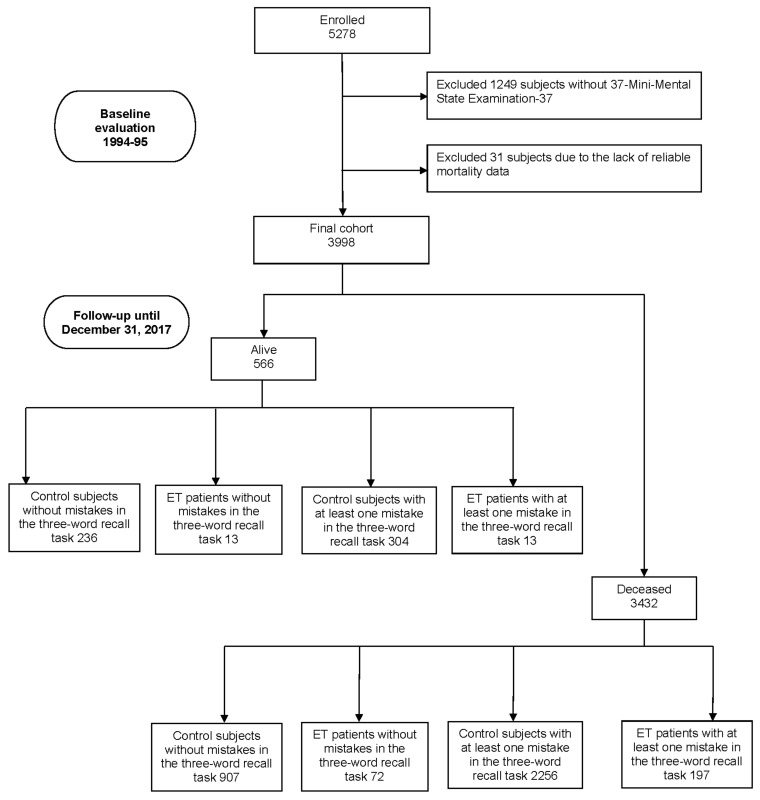
Flow chart of this study.

Of the 5278 participants screened for neurological conditions at baseline (1994–1995), 1249 (27.7%) had no MMSE-37 (including 34 prevalent ET cases—participants diagnosed with ET at baseline—and 8 ET premotor cases—participants identified with ET during the follow-up period but not at baseline—and 1207 controls). Additionally, 31 participants (0.6%) (one prevalent ET case, one ET premotor case, and 29 controls) were excluded due to the lack of reliable mortality data, leaving 3998 participants included in our analyses (Figure 1). A comparison between the 3998 included participants and the 1280 excluded individuals revealed that the included group was younger (73.7 ± 6.7 years; median = 72) compared to those excluded (75.5 ± 7.7 years; median = 74) (Mann–Whitney test, *p* < 0.001). Additionally, the proportion of women was lower among included participants (51.2%, n = 2047) than among those excluded (55.0%, n = 704) (chi-square [X^2^] = 5.61, *p* = 0.018). Furthermore, included participants had a higher level of education, with 14.1% (n = 562) having at least secondary education, compared to 11.8% (n = 146) in the excluded group (X^2^ = 31.35, *p* < 0.001).

In the current study, we included premotor ET patients—those first diagnosed with ET at follow-up (1997–1998)—as part of the ET patient group. This approach aligns with emerging research suggesting that non-motor symptoms may represent an early or variant stage of motor ET [12,17,45]. Previous studies support the idea of a progression from non-motor to motor symptoms in ET, indicating a continuum in the disease’s manifestation [12,45]. Therefore, including premotor ET patients in the ET group is justified for the analysis, as it reflects the evolving understanding of ET’s progression and spectrum [12,45].

Of the 3998 participants, 221 were identified as prevalent ET cases, 74 as ET premotor cases, and 3703 as controls (participants who did not meet the criteria for ET during either the baseline screening or the follow-up period) [36,37]. Among the 221 prevalent ET cases, three (1.4%) had isolated head tremor, eight (3.6%) presented with both limb and voice tremor, 18 (8.1%) exhibited head and limb tremor, three (1.4%) had head, limb, and voice tremor, and the remaining 189 (85.5%) had isolated limb tremor. None of them exhibited other neurological signs, such as dystonic posturing, that would not suffice to make an additional syndrome diagnosis.

### 2.5. Statistical Analyses

The statistical analyses for this study were conducted using SPSS software, version 29.0, and Python 3.12.2, with the packages pandas 2.2.2 and lifelines 0.29.0. All *p*-values were two-tailed, and we considered *p* < 0.05 significant. Even after log transformation, continuous variables were not normally distributed (Kolmogorov–Smirnov test, *p* < 0.05). Therefore, we used Mann–Whitney or Kruskal–Wallis tests to analyze these continuous variables, whereas the chi-square test or Fisher’s exact test, when the expected frequencies in any cell were less than five, was used to analyze categorical variables.

Using the Cox proportional hazards models, we calculated the mortality hazard ratios (HRs) and their 95% confidence intervals (CIs). The time variable covered the period from the baseline assessment (1994–1995) to 31 December 2017, or the date of death, whichever came first.

We categorized participants based on their performance in the three-word recall task. They were divided into four groups: control subjects without mistakes in the three-word recall task (reference category in the Cox proportional hazards models), ET patients without mistakes in the three-word recall task, control subjects with at least one mistake, and ET patients with at least one mistake. This classification allowed for a nuanced analysis of episodic memory performance across different groups, considering ET’s presence and the memory task’s accuracy. In each analysis, we began with an unadjusted model. In adjusted Cox proportional hazards analyses, model 1 used a more restrictive approach considering all baseline variables that were associated (*p* < 0.05) with both the groups of exposure (controls and ET patients, stratified by the performance of the three-word recall task) and the outcome (death). Next, we included baseline variables associated with either the exposure or the outcome in bivariate analyses (*p* < 0.05) using a less restrictive approach (model 2). Our comprehensive approach also adjusted for all potential confounding factors in the analysis, irrespective of their statistical correlation with the exposure or outcome variables. This adjustment was made to ensure the robustness of our findings, encompassing factors that might not show a direct link but could influence the results (model 3).

Age (years), sex, educational level, sleep duration, smoker (ever-smoker vs. never), consumption of ethanol (ever at least once per week vs. never), comorbidity index, arterial hypertension, and medications with potential cognitive effects were assessed at baseline and considered as potential covariates. 

Kaplan–Meier survival curves were utilized to evaluate and compare the survival of ET patients and non-ET subjects based on their performance in the MMSE-37’s three-word recall task. These groups were further divided based on whether they made any mistakes in the task. We used the log-rank test to compare the differences between the four survival curves, providing insights into the impact of ET and episodic memory performance on survival rates.

Additive interactions were assessed using the Relative Excess Risk due to Interaction (RERI), which measures deviation from additivity on the risk scale. A RERI of 0 indicates no interaction, while values above 0 suggest a positive interaction (combined effect exceeds the sum of individual effects), and values below 0 indicate a negative interaction (effect less than additivity) [46].

## 3. Results

Of the 3998 participants, 3432 (85.8%) died over a median follow-up of 11.2 years (mean = 11.5 years; range = 0.03–23.9 years). This included 907 deaths (79.3%) among the 1143 control subjects without mistakes in the three-word recall task, 72 deaths (84.7%) among the 85 ET patients without mistakes, 2256 deaths (88.1%) among the 2560 control subjects with at least one mistake, and 197 deaths (93.8%) among the 210 ET patients with at least one mistake (Figure 1). Notably, 210 out of 295 (71.2%) ET patients demonstrated memory impairments, aligning with the criteria for ET-plus [47], which encompasses additional neurological features such as cognitive deficits.

We observed demographic and clinical characteristics differences among participants divided by ET status and cognitive function (measured by a three-word recall task). Specifically, variations were observed in age and educational attainment, with ET patients and those with recall task errors tending to be older and possessing lower education levels. Additionally, lifestyle factors such as sleep duration, drinking, and smoking, as well as medical comorbidities, differed significantly among the groups (Table 1). Furthermore, ET patients were more likely to use medications with potential cognitive effects, regardless of their episodic memory status (Table 1).

Consistent with expectations, the analysis revealed that deceased individuals were notably older, with lower levels of education, longer sleep durations, a higher prevalence of smoking, and more medical comorbidities compared with their living counterparts, as detailed in Table 2. Additionally, deceased individuals were more likely to use medications with potential cognitive effects.

In an unadjusted Cox model, the mortality risk was increased in ET patients with at least one mistake in the three-word recall task (HR = 1.65, 95% CI = 1.41–1.93, *p* < 0.001) and controls with at least one mistake (HR = 1.42, 95% CI = 1.32–1.54, *p* < 0.001), but not in ET patients without any mistake (HR = 1.15, 95% CI = 0.91–1.47, *p* = 0.239) vs. those controls without any mistake (reference group). In a Cox regression model that incorporated adjustments for age in years, educational level, sleep duration, ever-smoker, comorbidity index, and medications with potential cognitive effects, i.e., variables that were associated with both the groups of exposure (controls and ET patients, stratified by the performance of the three-word recall task) and the outcome (death), the risk of mortality remained increased in ET patients with at least one mistake in the three-word recall task (HR = 1.26, 95% CI = 1.07–1.48, *p* = 0.004) and controls with at least one mistake (HR = 1.19, 95% CI = 1.10–1.29, *p* < 0.001), model 1 in Table 3.

The findings remained consistent in a Cox regression model even after adjusting for variables that were linked with either the groups of exposure and the outcome (death) (baseline age in years, educational level, sleep duration, ever-smoker, ever-drinker, comorbidity index, arterial hypertension, and medications with potential cognitive effects) (model 2 in Table 3). The results were similar in a Cox model that adjusted for all the potential confounders (baseline age in years, sex, educational level, sleep duration, ever-smoker, ever-drinker, comorbidity index, arterial hypertension, and medications with potential cognitive effects), independent of their statistical significance model 3 in Table 3).

**Figure 2:**
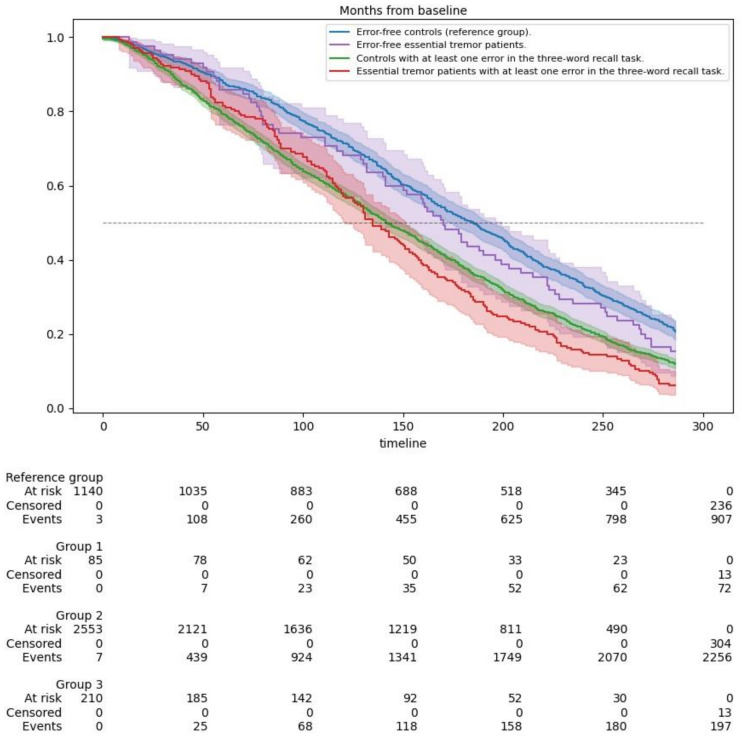
Kaplan–Meier survival curves for mortality risk in essential tremor patients and controls, stratified by performance on the three-word recall task (Log-Rank [Mantel–Cox] test: χ^2^ = 93.827, *p* < 0.001).

Mean survival times were derived from Kaplan–Meier estimates based on the baseline assessment (1994–1995). Figure 2 illustrates the survival curves for the study groups, stratified by disease status (controls and ET patients) and performance on the three-word recall task. A lower survival rate was observed in groups with episodic memory impairments, as evidenced by the Log-Rank (Mantel–Cox) test (χ^2^ = 93.827, *p* < 0.001). ET patients who made at least one mistake in the three-word recall task had slightly shorter survival times, with a median survival of 11.3 years (95% CI: 10.1–12.4) and a mean survival of 12.0 years (95% CI: 11.2–12.9), compared with ET patients without mistakes and controls (Table 4)

When we limited our analyses to the 1249 participants without MMSE-37 data, we observed no increased mortality risk among ET patients (unadjusted HR = 0.89, 95% CI = 0.64–1.23, *p* = 0.479) compared with controls (reference group). This finding remained consistent across all adjusted models (1, 2, and 3).

In other analyses, we first excluded the dementia cases, and the results did not change (Table 5), as did when we excluded the premotor ET patients (Table 6).

ET patients with memory impairments had a significantly higher AD-related mortality rate (5.1%) compared with those without memory impairments (1.4%, *p* = 0.013). Similarly, controls with memory impairments also exhibited increased AD-related mortality (5.1%) compared with controls without memory impairments (2.7%). No significant differences were observed between ET patients and controls in mortality rates for other primary causes of death, such as cerebrovascular disorders, cardiovascular diseases, respiratory diseases, or cancer. These findings highlight the pivotal role of memory impairment in AD-related mortality, with ET status possibly amplifying this risk but not independently driving the association.

An additive interaction between ET and memory impairment was assessed using the RERI. The unadjusted RERI was 0.08 (95% CI: −0.22–0.38), and the fully adjusted RERI was 0.11 (95% CI: −0.19–0.41).

## 4. Discussion

ET is increasingly recognized for its substantial phenotypic heterogeneity, encompassing both motor and non-motor manifestations. Beyond cognitive deficits, patients often exhibit olfactory dysfunction, psychiatric symptoms, personality changes, and sleep disturbances, reflecting a broader neurodegenerative process [25,26,48,49,50].

Our study revealed that episodic memory impairments, assessed through errors in a word recall task, were significantly associated with increased mortality risk across all models. In the fully adjusted model (model 3), ET patients with memory impairment had a moderately elevated hazard of mortality (HR = 1.25, 95% CI: 1.06–1.46, *p* = 0.007) compared with controls with memory impairment (HR = 1.19, 95% CI: 1.09–1.28, *p* < 0.001). Among those without memory impairments, ET patients did not exhibit a significant increase in mortality risk compared with controls (HR = 0.95, 95% CI: 0.74–1.21, *p* = 0.661).

The Kaplan–Meier survival curves showed that ET patients with episodic memory impairments had slightly lower survival trajectories than controls with similar memory deficits. This suggests that episodic memory impairment, rather than ET itself, is the primary driver of increased mortality risk, with ET possibly modifying or amplifying this effect.

To further explore the potential interaction between ET and episodic memory impairment, we assessed additive interaction using the RERI. The RERI values in both the unadjusted (0.08, 95% CI: −0.22 to 0.38) and fully adjusted models (0.11, 95% CI: −0.19 to 0.41) were not statistically significant, as both CIs included zero. These results indicate that we found no clear evidence of an additive interaction between ET and memory impairment. However, the wide confidence intervals suggest that this study may have been underpowered to detect subtle interactions, and a small effect cannot be entirely ruled out.

These findings suggest that ET and memory impairment contribute independently to mortality risk, with no strong evidence of an additive interaction. However, given the limitations of sample size and statistical power, further research with larger cohorts is needed to elucidate better the interplay between ET, cognitive impairment, and mortality risk. Future studies should consider alternative statistical approaches, such as multiplicative interaction models, Bayesian inference, or machine learning-based analyses, to explore potential synergistic effects that traditional RERI analyses may not capture.

The association between episodic memory impairments and increased mortality in ET patients warrants careful consideration, as it reflects the interplay of neurodegenerative processes, health management challenges, and functional decline. There is growing evidence suggesting that ET is associated with an increased likelihood of neurodegenerative diseases such as AD [14,15,48,49,50], which prominently features episodic memory loss. Cognitive impairments in ET, particularly episodic memory deficits, may signal the early stages of neurodegenerative processes that elevate mortality risk over time. This link is further supported by postmortem studies demonstrating a higher incidence of AD-type pathological changes in ET patients [19,20].

The pathophysiological overlap between ET and other neurodegenerative disorders remains a critical area of investigation. Inflammatory pathways, neurotransmitter dysregulation—particularly involving orexin—and neuroimmune activation have been implicated in the pathogenesis of Parkinson’s disease [51] and AD [52], suggesting similar mechanisms may contribute to ET-related neurodegeneration. Inflammation, in particular, plays a central role in neuronal dysfunction, synaptic loss, and disease progression across multiple neurodegenerative conditions [51,52,53]. Future research should determine whether ET represents an early-stage neurodegenerative process with shared inflammatory and neuroimmune pathways, which could guide the development of targeted therapies.

Genetic factors significantly contribute to ET, with over 50% of affected individuals having a positive family history [54]. Several susceptibility genes have been proposed, including LINGO1, SLC1A2, STK32B, PPARGC1A, and CTNNA3, though none have been consistently confirmed across studies [54,55]. The genetic architecture of ET appears complex, involving variable penetrance and polygenic influences [54,55]. Given the substantial phenotypic heterogeneity of ET, future research should explore whether familial ET represents a genetically distinct subgroup with differing cognitive trajectories and mortality risks compared with sporadic cases.

Emerging theories on cognitive impairment in ET emphasize cerebellar dysfunction, drawing parallels with the cerebellar cognitive affective syndrome as a reference for understanding deficits in attention, executive function, and language [56,57]. Additionally, ET affects multiple brain regions beyond those controlling tremor [58,59,60], with evidence of frontal lobe dysfunction pointing to a complex interaction between cerebellar–thalamocortical pathways and higher-order processes [7,8,9,58,59,60,61]. Understanding these mechanisms may inform therapeutic strategies, with interventions targeting cerebellar dysfunction and modulating these pathways offering potential avenues to mitigate cognitive decline and reduce mortality in ET patients [60].

Finally, memory impairment can adversely affect health management by compromising medication adherence, symptom recognition, and compliance with medical recommendations, leading to increased complications and poorer outcomes. Cognitive decline also contributes to functional impairments, greater frailty, and increased susceptibility to injuries and infections, further elevating mortality risk [62].

This study has limitations. First, while the MMSE-37’s three-word recall task is a widely used and practical measure of episodic memory in large epidemiological studies [1,5,6], it may lack the sensitivity and specificity of more comprehensive neuropsychological assessments. The reliance on this single task might have led to misclassification of episodic memory impairment, potentially underestimating its true impact on mortality risk. Future studies should incorporate more robust and validated episodic memory tests, such as the Free and Cued Selective Reminding Test (FCSRT), to enhance the precision of cognitive assessments. Additionally, reassessment using a more comprehensive cognitive screening tool, such as the Montreal Cognitive Assessment (MoCA), could provide further validation of our findings. Notwithstanding, adjustments for confounders such as dementia, psychiatric disorders, and educational level likely mitigated inaccuracies associated with this measure.

Second, we excluded participants without MMSE-37 data. However, similar mortality risks in controls and ET cases without MMSE-37 data suggest these exclusions likely did not bias conclusions on the association between ET, episodic memory impairment, and mortality.

Thirdly, despite the rigorous consensus process involving three neurologists at baseline and follow-up to classify ET cases, some misclassification is inevitable, especially in long-term studies where conditions like Parkinson’s disease or dystonia may emerge. ET’s association with other neurodegenerative diseases, such as Parkinson’s and progressive supranuclear palsy [63,64], further complicates diagnosis. Future research using advanced diagnostic tools, including biomarkers or postmortem analyses, could improve diagnostic accuracy and understanding of ET’s progression and comorbidities.

Additionally, our reliance on death certificates to determine AD-related mortality introduces potential inaccuracies [65], as clinical AD diagnoses are prone to misclassification without biomarkers or autopsy data [66,67]. Other neurodegenerative diseases among deceased participants were not systematically accounted for, raising the possibility of coexisting or alternative conditions. While we reviewed clinical records to validate causes of death, the lack of neuropathological confirmation or biomarkers leaves some diagnostic uncertainty.

Finally, this study focuses on a Spanish cohort, which may limit the generalizability of the findings to other populations. Differences in genetic background, healthcare systems, and environmental factors could influence the observed associations. Conducting similar studies in diverse populations will be essential to validate these findings and determine their broader applicability.

Despite these limitations, this study has several strengths. Firstly, the population-based design enabled the assessment of a broad, unselected group of older adults. Secondly, the standardized and prospective nature of the assessments reduced potential biases. Lastly, this study accounted for the confounding effects of various critical factors, enhancing the reliability of its findings.

In summary, this study underscores the critical role of episodic memory impairment in influencing health outcomes among older adults with ET. Given that episodic memory deficits are a hallmark of AD, they may indicate overlapping pathophysiological mechanisms or imply that ET contributes to heightened cognitive susceptibility. Findings from the NEDICES study show that while ET does not universally increase mortality risk, it is significantly higher in ET patients with episodic memory impairments. This underscores the need for further research into the mechanisms linking ET to cognitive networks and memory, which could enhance understanding of its neurological impact and guide targeted interventions.

## Figures and Tables

**Table 1:** Baseline demographic and clinical characteristics of the cohort stratified by disease status (essential tremor vs. control) and performance in the three-word recall task (N = 3998).

	Control Subjects without Mistakes in the Three-Word Recall Task (N = 1143)	ET Patients without Mistakes in the Three-Word Recall Task (N = 85)	Control Subjects with at Least One Mistake (N = 2560)	ET Patients with at Least One Mistake (N = 210)	*p*-Value
Age (years)	71.8 (70.0) ± 5.8	73.0 (72.0) ± 6.4	74.5 (73.0) ± 6.9	75.4 (75.0) ± 7.0	<0.001 ^a^
Sex (women)	582 (50.9%)	41 (48.2%)	1313 (51.3%)	111 (52.9%)	0.902 ^b^
Educational level					<0.001 ^b^
*Illiterate*	114 (10.0%)	18 (21.2%)	368 (14.4%)	41 (19.5%)	
*Can read and write*	439 (38.4%)	30 (35.3%)	1099 (42.9%)	89 (42.4%)	
*Primary studies*	363 (31.8%)	24 (28.2%)	797 (31.1%)	54 (25.7%)	
*Secondary and higher studies*	227 (19.9%)	13 (15.3%)	296 (11.6%)	26 (12.4%)	
Sleep duration (hours per day) *					<0.001 ^b^
*≤5*	93 (8.2%)	8 (9.5%)	288 (11.4%)	38 (18.4%)	
*6–8*	564 (49.9%)	44 (52.4%)	1166 (46.0%)	93 (45.1%)	
*≥9*	473 (41.9%)	32 (38.1%)	1077 (42.6%)	75 (36.4%)	
Ever-smoker (ex-smoker plus current smoker) *	487 (42.7%)	36 (42.4%)	970 (37.9%)	69 (32.9%)	0.009 ^b^
Ever-drinker (ex-drinker plus current drinker) *	660 (57.8%)	49 (57.6%)	1360 (53.2%)	107 (51.0%)	0.041 ^b^
Comorbidity index ¥	0.9 (0.0) ± 1.2	1.3 (1.0) ± 1.6	1.2 (0.5) ± 1.6	1.3 (1.0) ± 1.6	<0.001 ^a^
Arterial hypertension *	559 (48.9%)	51 (60.0%)	1331 (52.1%)	114 (54.5%)	0.089 ^b^
Medications with potential cognitive effects	178 (15.6%)	24 (28.2%)	409 (16.0%)	54 (25.7%)	<0.001 ^b^
Dementia	5 (0.4%)	1 (1.2%)	159 (6.2%)	12 (5.7%)	<0.001 ^b^

^a^ Kruskal–Wallis test; ^b^ chi-square test or Fisher’s *p*-test when appropriate. Mean (median) ± standard deviation and frequency (%) are reported. * Data on some participants were missing. ¥ The comorbidity index is a comprehensive measure that accounts for the presence of multiple conditions, including atrial fibrillation, non-metastatic cancer, metastatic cancer, chronic obstructive pulmonary disease, depression, dementia, diabetes, epilepsy (treated), heart failure, myocardial infarction, psychiatric disorders, renal disease, and stroke.

**Table 2:** Baseline demographic and clinical characteristics of the cohort (essential tremor and controls) stratified by mortality (N = 3998).

	Deceased (N = 3432)	Alive (N = 566)	*p*-Value
Age (years)	74.6 (73.0) ± 6.7	68.7 (68.0) ± 3.6	<0.001 ^a^
Sex (women)	1736 (50.6%)	327 (54.8%)	0.059 ^b^
Educational level *			0.004 ^b^
*Illiterate*	486 (14.2%)	56 (9.7%)	
*Can read and write*	1419 (41.3%)	238 (42.0%)	
*Primary studies*	1065 (31.0%)	173 (30.6%)	
*Secondary and higher studies*	462 (13.5%)	100 (17.7%)	
Sleep duration (hours per day) *			<0.001 ^b^
*≤5*	374 (11.0%)	53 (9.4%)	
*6–8*	1549 (45.7%)	318 (56.6%)	
*≥9*	1466 (43.3%)	191 (34.0%)	
Ever-smoker (ex-smoker plus current smoker) *	1377 (40.2%)	185 (32.7%)	<0.001 ^b^
Ever-drinker (ex-drinker plus current drinker) *	1871 (54.6%)	305 (53.9%)	0.748 ^b^
Comorbidity index ¥	1.2 (1.0) ± 1.6	0.6 (0.0) ± 1.1	<0.001 ^a^
Arterial hypertension *	1839 (53.6%)	216 (38.2%)	<0.001 ^b^
Medications with potential cognitive effects	589 (17.2%)	76 (13.4)	0.027 ^b^
Dementia	176 (5.1%)	1 (0.2%)	<0.001 ^b^

^a^ Mann–Whitney test; ^b^ chi-square test or Fisher’s *p*-test when appropriate. Mean (median) ± standard deviation and frequency (%) are reported. * Data on some participants were missing. ¥ The comorbidity index is a comprehensive measure that accounts for the presence of multiple conditions, including atrial fibrillation, non-metastatic cancer, metastatic cancer, chronic obstructive pulmonary disease, depression, dementia, diabetes, epilepsy (treated), heart failure, myocardial infarction, psychiatric disorders, renal disease, and stroke.

**Table 3:** Mortality risks in participants stratified by disease status (essential tremor vs. control) and performance in the three-word recall task (N = 3998).

	Unadjusted			Model 1			Model 2			Model 3		
	Hazard Ratio	95% CI	*p*-Value	Hazard Ratio	95% CI	*p*-Value	Hazard Ratio	95% CI	*p*-Value	Hazard Ratio	95% CI	*p*-Value
Essential tremor patients with at least one mistake (N = 210)	1.65	1.41–1.93	<0.001	1.26	1.07–1.48	0.004	1.24	1.06–1.46	0.007	1.25	1.06–1.46	0.007
Control subjects with at least one mistake (N = 2560)	1.42	1.32–1.54	<0.001	1.19	1.10–1.29	<0.001	1.18	1.09–1.28	<0.001	1.19	1.09–1.28	<0.001
Essential tremor patients without mistakes in the three-word recall task (N = 85)	1.15	0.91–1.47	0.239	1.00	0.78–1.28	0.989	0.94	0.74–1.21	0.648	0.95	0.74–1.21	0.661
Control subjects without mistakes in the three-word recall task (N = 1143) (reference category)	1.00	_		1.00	_	_	1.00	_	_	1.00	_	

Model 1: Adjusted for baseline age (years), educational level, sleep duration, ever-smoker, comorbidity index, and medications with potential cognitive effects. Model 2: Adjusted for baseline age (years), educational level, sleep duration, ever-smoker, ever-drinker, comorbidity index, arterial hypertension, and medications with potential cognitive effects. Model 3: Adjusted for baseline age (years), sex, educational level, sleep duration, ever-smoker, ever-drinker, comorbidity index, arterial hypertension, and medications with potential cognitive effects.

**Table 4:** Median and mean survival time in years, according to disease status (essential tremor vs. control) and performance in the three-word recall task.

	Survival in Years (Median, 95% Confidence Interval)	Survival in Years (Mean, 95% Confidence Interval)
Control subjects without mistakes in the three-word recall task	15.5 (14.6–16.3)	15.0 (14.6–15.4)
Essential tremor patients without mistakes in the three-word recall task	14.2 (12.6–15.9)	14.3 (12.8–15.8)
Control subjects with at least one mistake	11.9 (11.4–12.3)	12.5 (12.2–12.8)
Essential tremor patients with at least one mistake	11.3 (10.1–12.4)	12.0 (11.2–12.9)

Log-Rank (Mantel–Cox) test: χ^2^ = 93.827, *p* < 0.001.

**Table 5:** Mortality risks in participants stratified by disease status (essential tremor vs. control) and performance in the three-word recall task, excluding 177 prevalent dementia cases (N = 3821).

	Unadjusted			Model 1			Model 2			Model 3		
	Hazard Ratio	95% CI	*p*-Value	Hazard Ratio	95% CI	*p*-Value	Hazard Ratio	95% CI	*p*-Value	Hazard Ratio	95% CI	*p*-Value
Essential tremor patients with at least one mistake (N = 200)	1.63	1.39–1.91	<0.001	1.36	1.16–1.60	<0.001	1.32	1.12–1.56	<0.001	1.32	1.12–1.55	<0.001
Control subjects with at least one mistake (N = 2420)	1.36	1.26–1.47	<0.001	1.18	1.09–1.27	<0.001	1.17	1.08–1.27	<0.001	1.17	1.08–1.27	<0.001
Essential tremor patients without mistakes in the three-word recall task (N = 84)	1.15	0.90–1.46	0.259	1.01	0.79–1.29	0.960	0.95	0.74–1.21	0.676	0.95	0.74–1.21	0.690
Control subjects without mistakes in the three-word recall task (N = 1147) (reference category)	1.00	_		1.00	_	_	1.00	_	_	1.00	_	

Model 1: Adjusted for baseline age (years), educational level, sleep duration, ever-smoker, comorbidity index, and medications with potential cognitive effects. Model 2: Adjusted for baseline age (years), educational level, sleep duration, ever-smoker, ever-drinker, comorbidity index, arterial hypertension, and medications with potential cognitive effects. Model 3: Adjusted for baseline age (years), sex, educational level, sleep duration, ever-smoker, ever-drinker, comorbidity index, arterial hypertension, and medications with potential cognitive effects.

**Table 6:** Mortality risks in participants stratified by disease status (essential tremor vs. control) and performance in the three-word recall task, excluding 74 premotor ET patients (N = 3924).

	Unadjusted			Model 1			Model 2			Model 3		
	Hazard Ratio	95% CI	*p*-Value	Hazard Ratio	95% CI	*p*-Value	Hazard Ratio	95% CI	*p*-Value	Hazard Ratio	95% CI	*p*-Value
Essential tremor patients with at least one mistake (N = 163)	1.77	1.49–2.10	<0.001	1.27	1.07–1.52	0.008	1.27	1.07–1.52	0.007	1.28	1.07–1.52	0.007
Control subjects with at least one mistake (N = 2580)	1.42	1.32–1.54	<0.001	1.19	1.10–1.29	<0.001	1.18	1.09–1.28	<0.001	1.18	1.09–1.28	<0.001
Essential tremor patients without mistakes in the three-word recall task (N = 59)	1.08	0.81–1.44	0.600	0.88	0.66–1.19	0.408	0.83	0.62–1.12	0.217	0.83	0.62–1.12	0.222
Control subjects without mistakes in the three-word recall task (N = 1152) (reference category)	1.00	_		1.00	_	_	1.00	_	_	1.00	_	

Model 1: Adjusted for baseline age (years), educational level, sleep duration, ever-smoker, comorbidity index, and medications with potential cognitive effects. Model 2: Adjusted for baseline age (years), educational level, sleep duration, ever-smoker, ever-drinker, comorbidity index, arterial hypertension, and medications with potential cognitive effects. Model 3: Adjusted for baseline age (years), sex, educational level, sleep duration, ever-smoker, ever-drinker, comorbidity index, arterial hypertension, and medications with potential cognitive effects.

## Data Availability

The original contributions presented in this study are included in the article. Further inquiries can be directed to the corresponding author.

## References

[1] Villarejo A., Bermejo-Pareja F., Trincado R., Olazarán J., Benito-León J., Rodríguez C., Medrano M.J., Boix R., Vega S. (2011). Memory impairment in a simple recall task increases mortality at 10 years in non-demented elderly. Int. J. Geriat. Psychiatry.

[2] Contador I., Bermejo-Pareja F., Mitchell A.J., Trincado R., Villarejo A., Sánchez-Ferro Á., Benito-León J. (2014). Cause of death in mild cognitive impairment: A prospective study (NEDICES). Euro. J. Neurol..

[3] Benito-León J., Contador I., Mitchell A.J., Domingo-Santos Á., Bermejo-Pareja F. (2016). Performance on Specific Cognitive Domains and Cause of Death: A Prospective Population-Based Study in Non-Demented Older Adults (NEDICES). J. Alzheimers Dis..

[4] Villarejo A., Benito-León J., Trincado R., Posada I.J., Puertas-Martín V., Boix R., Medrano M.J., Bermejo-Pareja F. (2011). Dementia-Associated Mortality at Thirteen Years in the NEDICES Cohort Study. J. Alzheimers Dis..

[5] Olazarán J., Trincado R., Bermejo F., Benito-León J., Díaz J., Vega S. (2004). Selective memory impairment on an adapted Mini-Mental State Examination increases risk of future dementia. Int. J. Geriat. Psychiatry.

[6] Carcaillon L., Amieva H., Auriacombe S., Helmer C., Dartigues J.-F. (2009). A Subtest of the MMSE as a Valid Test of Episodic Memory? Comparison with the Free and Cued Reminding Test. Dement. Geriatr. Cogn. Disord..

[7] Benito-León J., Louis E.D., Bermejo-Pareja F. (2006). Population-based case-control study of cognitive function in essential tremor. Neurology.

[8] Puertas-Martín V., Villarejo-Galende A., Fernández-Guinea S., Romero J.P., Louis E.D., Benito-León J. (2016). A Comparison Study of Cognitive and Neuropsychiatric Features of Essential Tremor and Parkinson’s Disease. Tremor Other Hyperkinetic Mov..

[9] Lafo J.A., Jones J.D., Okun M.S., Bauer R.M., Price C.C., Bowers D. (2015). Memory Similarities Between Essential Tremor and Parkinson’s Disease: A Final Common Pathway?. Clin. Neuropsychol..

[10] Louis E.D., Benito-Leon J., Vega-Quiroga S., Bermejo-Pareja F. (2010). The Neurological Disorders in Central Spain (NEDICES) Study Group. Cognitive and motor functional activity in non-demented community-dwelling essential tremor cases. J. Neurol. Neurosurg. Psychiatry.

[11] Louis E.D., Benito-León J., Vega-Quiroga S., Bermejo-Pareja F. (2010). the Neurological Disorders in Central Spain (NEDICES) Study Group. Faster rate of cognitive decline in essential tremor cases than controls: A prospective study. Euro. J. Neurol..

[12] Benito-León J., Louis E.D., Sánchez-Ferro Á., Bermejo-Pareja F. (2013). Rate of cognitive decline during the premotor phase of essential tremor: A prospective study. Neurology.

[13] Benito-León J., Louis E.D., Mitchell A.J., Bermejo-Pareja F. (2011). Elderly-Onset Essential Tremor and Mild Cognitive Impairment: A Population-Based Study (NEDICES). J. Alzheimers Dis..

[14] Benito-León J., Louis E.D., Bermejo-Pareja F. (2006). Elderly-onset essential tremor is associated with dementia. Neurology.

[15] Bermejo-Pareja F., Louis E.D., Benito-León J. (2007). Risk of incident dementia in essential tremor: A population-based study. Mov. Disord..

[16] Thawani S.P., Schupf N., Louis E.D. (2009). Essential tremor is associated with dementia: Prospective population-based study in New York. Neurology.

[17] Benito-León J., Contador I., Louis E.D., Cosentino S., Bermejo-Pareja F. (2016). Education and risk of incident dementia during the premotor and motor phases of essential tremor (NEDICES). Medicine.

[18] Radler K.H., Zdrodowska M.A., Dowd H., Cersonsky T.E., Huey E.D., Cosentino S., Louis E.D. (2020). Rate of progression from mild cognitive impairment to dementia in an essential tremor cohort: A prospective, longitudinal study. Park. Relat. Disord..

[19] Béliveau E., Tremblay C., Aubry-Lafontaine É., Paris-Robidas S., Delay C., Robinson C., Ferguson L., Rajput A.H., Rajput A., Calon F. (2015). Accumulation of amyloid-β in the cerebellar cortex of essential tremor patients. Neurobiol. Dis..

[20] Farrell K., Cosentino S., Iida M.A., Chapman S., A Bennett D., Faust P.L., Louis E.D., Crary J.F. (2019). Quantitative Assessment of Pathological Tau Burden in Essential Tremor: A Postmortem Study. J. Neuropathol. Exp. Neurol..

[21] Rajput A.H., Offord K.P., Beard C.M., Kurland L.T. (1984). Essential tremor in Rochester, Minnesota: A 45-year study. J. Neurol. Neurosurg. Psychiatry.

[22] Louis E.D., Benito-León J., Ottman R., Bermejo-Pareja F. (2007). A population-based study of mortality in essential tremor. Neurology.

[23] Delgado N., Hernandez D.I., Radler K., Huey E.D., Cosentino S., Louis E. (2021). Mild cognitive impairment, dementia and risk of mortality in essential tremor: A longitudinal prospective study of elders. J. Neurol. Sci..

[24] Zubair A., Cersonsky T.E.K., Kellner S., Huey E.D., Cosentino S., Louis E.D. (2018). What Predicts Mortality in Essential Tremor? A Prospective, Longitudinal Study of Elders. Front. Neurol..

[25] Benito-León J., Louis E.D. (2006). Essential tremor: Emerging views of a common disorder. Nat. Clin. Pract. Neurol..

[26] Benito-León J., Louis E.D. (2011). Update on essential tremor. Minerva Med..

[27] Morales J.M., Bermejo F.P., Benito-León J., Rivera-Navarro J., Trincado R., Gabriel S.R., Vega S. (2004). Methods and demographic findings of the baseline survey of the NEDICES cohort: A door-to-door survey of neurological disorders in three communities from Central Spain. Public Health.

[28] Bermejo-Pareja F., Benito-León J., Vega-Q S., Díaz-Guzmán J., Rivera-Navarro J., Molina J.A., Olazarán-Rodríguez J., Morales-González J.M. (2008). La cohorte de ancianos NEDICES. Metodología y principales hallazgos neurológicos [The NEDICES cohort of the elderly. Methodology and main neurological findings]. Rev. Neurol..

[29] Vega S., Benito-León J., Bermejo-Pareja F., Medrano M.J., Vega-Valderrama L.M., Rodríguez C., Louis E.D. (2010). Several factors influenced attrition in a population-based elderly cohort: Neurological disorders in Central Spain Study. J. Clin. Epidemiol..

[30] Serna A., Contador I., Bermejo-Pareja F., Mitchell A.J., Fernández-Calvo B., Ramos F., Villarejo A., Benito-León J. (2015). Accuracy of a Brief Neuropsychological Battery for the Diagnosis of Dementia and Mild Cognitive Impairment: An Analysis of the NEDICES Cohort. J. Alzheimers Dis..

[31] Contador I., Bermejo-Pareja F., Fernández-Calvo B., Boycheva E., Tapias E., Llamas S., Benito-León J. (2016). The 37 item Version of the Mini-Mental State Examination: Normative Data in a Population-Based Cohort of Older Spanish Adults (NEDICES). Arch. Clin. Neuropsychol..

[32] Folstein M.F., Folstein S.E., McHugh P.R. (1975). “Mini-mental state.” A practical method for grading the cognitive state of patients for the clinician. J. Psychiatr. Res..

[33] Carey I.M., Shah S.M., Harris T., DeWilde S., Cook D.G. (2013). A new simple primary care morbidity score predicted mortality and better explains between practice variations than the Charlson index. J. Clin. Epidemiol..

[34] Benito-León J., Louis E.D., Bermejo-Pareja F. (2013). Cognitive decline in short and long sleepers: A prospective population-based study (NEDICES). J. Psychiatr. Res..

[35] Benito-León J., Louis E.D., Villarejo-Galende A., Romero J.P., Bermejo-Pareja F. (2014). Long sleep duration in elders without dementia increases risk of dementia mortality (NEDICES). Neurology.

[36] Benito-León J., Bermejo-Pareja F., Morales J., Vega S., Molina J. (2003). Prevalence of essential tremor in three elderly populations of central Spain. Mov. Disord..

[37] Benito-León J., Bermejo-Pareja F., Louis E.D. (2005). Incidence of essential tremor in three elderly populations of central Spain. Neurology.

[38] Bermejo F., Gabriel R., Vega S., Morales J.M., Rocca W.A., Anderson D.W. (2001). Problems and Issues with Door-To-Door, Two-Phase Surveys: An Illustration from Central Spain. Neuroepidemiology.

[39] Salemi G., Savettieri G., Rocca W.A., Meneghini F., Saporito V., Morgante L., Reggio A., Grigoletto F., Di Perri R. (1994). Prevalence of essential tremor: A door-to-door survey in Terrasini, Sicily. Neurology.

[40] Salemi G., Aridon P., Calagna G., Monte M., Savettieri G. (1998). Population-based case-control study of essential tremor. Ital. J. Neurol. Sci..

[41] Benito-León J., León-Ruiz M. (2020). Epidemiología del temblor esencial [Epidemiology of essential tremor]. Rev. Neurol..

[42] American Psychiatric Association (1994). AP Association Task Force on DSM-IV. Diagnostic and Statistical Manual of Mental Disorders: DSM-IV.

[43] Bermejo-Pareja F., Benito-León J., Vega S., Olazarán J., de Toledo M., Díaz-Guzmán J., Sánchez-Sánchez F., Morales-González J., Trincado R., Portera-Sánchez A. (2009). Consistency of clinical diagnosis of dementia in NEDICES: A population-based longitudinal study in Spain. J. Geriatr. Psychiatry Neurol..

[44] Bermejo-Pareja F., Benito-León J., Vega S., Medrano M.J., Román G.C. (2008). Neurological Disorders in Central Spain (NEDICES) Study Group. Incidence and subtypes of dementia in three elderly populations of central Spain. J. Neurol. Sci..

[45] Lenka A., Benito-León J., Louis E.D. (2017). Is there a Premotor Phase of Essential Tremor?. Tremor Other Hyperkinetic Mov..

[46] Li R., Chambless L. (2007). Test for additive interaction in proportional hazards models. Ann. Epidemiol..

[47] Bhatia K.P., Bain P., Bajaj N., Elble R.J., Hallett M., Louis E.D., Raethjen J., Stamelou M., Testa C.M., Deuschl G. (2018). Consensus Statement on the classification of tremors. from the task force on tremor of the International Parkinson and Movement Disorder Society. Mov. Disord..

[48] Benito-León J. (2011). Essential Tremor: One of the Most Common Neurodegenerative Diseases?. Neuroepidemiology.

[49] Benito-León J. (2014). Essential Tremor: A Neurodegenerative Disease?. Tremor Other Hyperkinetic Mov..

[50] Bermejo-Pareja F., Benito-León J. (2010). El temblor esencial es una enfermedad neurodegenerativa [Essential tremor is a neurodegenerative disease]. Med. Clin.

[51] McGeer P.L., McGeer E.G. (2004). Inflammation and neurodegeneration in Parkinson’s disease. Park. Relat. Disord..

[52] McGeer E.G., McGeer P.L. (1998). The importance of inflammatory mechanisms in Alzheimer disease. Exp. Gerontol..

[53] Alster P., Madetko-Alster N., Otto-Ślusarczyk D., Migda A., Migda B., Struga M., Friedman A. (2023). Role of orexin in pathogenesis of neurodegenerative parkinsonisms. Neurol. Neurochir. Pol..

[54] Tio M., Tan E.K. (2016). Genetics of essential tremor. Park. Relat. Disord..

[55] Jiménez-Jiménez F.J., Alonso-Navarro H., García-Martín E., Álvarez I., Pastor P., Agúndez J.A.G. (2021). Genomic Markers for Essential Tremor. Pharmaceuticals.

[56] Benito-León J., Labiano-Fontcuberta A. (2016). Linking Essential Tremor to the Cerebellum: Clinical Evidence. Cerebellum.

[57] Ghosh R., León-Ruiz M., Sardar S.S., Naga D., Ghosh T., Dutta S., Benito-León J. (2022). Cerebellar Cognitive Affective Syndrome in a Case of Cerebrotendinous Xanthomatosis. Cerebellum.

[58] Benito-León J., Louis E.D., Romero J.P., Hernández-Tamames J.A., Manzanedo E., Álvarez-Linera J., Bermejo-Pareja F., Posada I., Rocon E. (2015). Altered Functional Connectivity in Essential Tremor: A Resting-State fMRI Study. Medicine.

[59] Benito-León J., Sanz-Morales E., Melero H., Louis E.D., Romero J.P., Rocon E., Malpica N. (2019). Graph theory analysis of resting-state functional magnetic resonance imaging in essential tremor. Hum. Brain Mapp..

[60] Younger E., Ellis E.G., Parsons N., Pantano P., Tommasin S., Caeyenberghs K., Benito-León J., Romero J.P., Joutsa J., Corp D.T. (2023). Mapping Essential Tremor to a Common Brain Network Using Functional Connectivity Analysis. Neurology.

[61] Cartella S.M., Bombaci A., Gallo G., Ledda C., Pengo M., Pignolo A., Pozzi F.E., Spina E., Trinchillo A., Palermo G. (2022). Essential tremor and cognitive impairment: Who, how, and why. Neurol. Sci..

[62] Aguilar-Navarro S.G., Mimenza-Alvarado A.J., Yeverino-Castro S.G., Caicedo-Correa S.M., Cano-Gutiérrez C. (2024). Cognitive Frailty and Aging: Clinical Characteristics, Pathophysiological Mechanisms, and Potential Prevention Strategies. Arch. Med. Res..

[63] Louis E.D., Iglesias-Hernandez D., Hernandez N.C., Flowers X., Kuo S.-H., Vonsattel J.P.G., Faust P.L. (2022). Characterizing Lewy Pathology in 231 Essential Tremor Brains from the Essential Tremor Centralized Brain Repository. J. Neuropathol. Exp. Neurol..

[64] Louis E.D., Babij R., Ma K., Cortés E., Vonsattel J.-P.G. (2013). Essential Tremor Followed by Progressive Supranuclear Palsy: Postmortem Reports of 11 Patients. J. Neuropathol. Exp. Neurol..

[65] Pritt B.S., Hardin N.J., Richmond J.A., Shapiro S.L. (2005). Death Certification Errors at an Academic Institution. Arch. Pathol. Lab. Med..

[66] Dhauria M., Mondal R., Deb S., Shome G., Chowdhury D., Sarkar S., Benito-León J. (2024). Blood-Based Biomarkers in Alzheimer's Disease: Advancing Non-Invasive Diagnostics and Prognostics. Int. J. Mol. Sci..

[67] Beach T.G., Monsell S.E., Phillips L.E., Kukull W. (2012). Accuracy of the Clinical Diagnosis of Alzheimer Disease at National Institute on Aging Alzheimer Disease Centers, 2005–2010. J. Neuropathol. Exp. Neurol..

